# Dopant-specific unzipping of carbon nanotubes for intact crystalline graphene nanostructures

**DOI:** 10.1038/ncomms10364

**Published:** 2016-01-22

**Authors:** Joonwon Lim, Uday Narayan Maiti, Na-Young Kim, Rekha Narayan, Won Jun Lee, Dong Sung Choi, Youngtak Oh, Ju Min Lee, Gil Yong Lee, Seok Hun Kang, Hyunwoo Kim, Yong-Hyun Kim, Sang Ouk Kim

**Affiliations:** 1 National Creative Research Initiative Center for Multi-Dimensional Directed Nanoscale Assembly, Department of Materials Science and Engineering, KAIST, Daejeon 34141, Republic of Korea; 2 Graduate School of Nanoscience and Technology, KAIST, Daejeon 34141, Republic of Korea; 3 Department of Chemistry, KAIST, Daejeon 34141, Republic of Korea

## Abstract

Atomic level engineering of graphene-based materials is in high demand to enable customize structures and properties for different applications. Unzipping of the graphene plane is a potential means to this end, but uncontrollable damage of the two-dimensional crystalline framework during harsh unzipping reaction has remained a key challenge. Here we present heteroatom dopant-specific unzipping of carbon nanotubes as a reliable and controllable route to customized intact crystalline graphene-based nanostructures. Substitutional pyridinic nitrogen dopant sites at carbon nanotubes can selectively initiate the unzipping of graphene side walls at a relatively low electrochemical potential (0.6 V). The resultant nanostructures consisting of unzipped graphene nanoribbons wrapping around carbon nanotube cores maintain the intact two-dimensional crystallinity with well-defined atomic configuration at the unzipped edges. Large surface area and robust electrical connectivity of the synergistic nanostructure demonstrate ultrahigh-power supercapacitor performance, which can serve for AC filtering with the record high rate capability of −85° of phase angle at 120 Hz.

## Abstract (web summary)

Atomic level engineering of graphene-based materials is highly demanded for the customized structures and properties. Here, the authors show heteroatom dopant-specific unzipping of carbon nanotubes as a reliable and controllable route to customized 'intact crystalline' graphene-based nanostructures.

## Graphical abstract

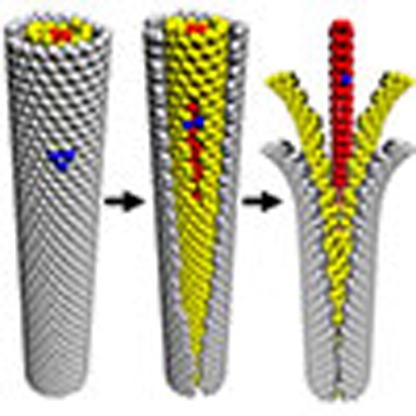

Unzipping of graphene basal plane is a valuable route to tailor carbon nanostructures^1^,^2^,^3^,^4^,^5^,^6^,^7^,^8^,^9^. Complete nanoscale unzipping of carbon nanotubes (CNTs) or graphene may produce graphene nanoribbons with electrical energy band gap^10^,^11^,^12^,^13^,^14^. Partial unzipping of CNTs may create unique nanostructures where CNTs and graphene nanoribbons are seamlessly connected^2^,^15^. Since the pioneering work by Tour *et al*.^1^, many different unzipping mechanisms have been explored. Wet chemical methods, including chemical oxidation^1^,^16^, Li ion intercalation^17^, sonication^18^, electrochemical method^19^ and hydrothermal reaction^20^ are advantageous for scalable solution processing. Dry processing techniques, such as plasma etching^12^, metal particle catalytic cutting^21^, rapid thermal expansion^22^ and high mechanical impact^23^ can reduce the oxidative degradation of unzipped structures. Unfortunately, harsh reaction conditions for the unzipping of robust sp^2^ hybridized graphene plane commonly accompanies undesired damage in the basal plane. A better controllability over unzipping mechanism is highly demanded for the minimal damage to the genuine graphene-based carbon structures^16^,^24^,^25^,^26^,^27^.

In this work, we present dopant-specific electrochemical unzipping of CNTs as a controllable method for intact crystalline unzipping. Heteroatom dopants, such as nitrogen substitutionally incorporated into sp^2^ hybridized carbon framework, enable atomic-scale site-selective unzipping reaction. Although pristine CNTs are stable up to the electrochemical potential above 0.8 V, nitrogen-doped CNTs (NCNTs) are readily unzipped below 0.6 V. Detailed investigation on the reaction mechanism reveals that substitutional pyridinic nitrogen (N_p_)-dopant can specifically initiate CNT wall unzipping. Such a dopant-specific unzipping at moderate potential enables fine controllability of unzipping level and intact crystallinity of the unzipped structures with well-defined edge configuration. Taking advantage of these unique features, we are able to synthesize intact crystalline graphene-based nanostructures, where unzipped graphene nanoribbons are seamlessly connected to the CNT strands. This structural feature offers synergistic properties comprising large surface area and robust electrical conductivity, which are highly desirable for electrochemical applications, such as energy storage. As a representative application, we demonstrate ultrahigh-power double-layer capacitors (DLCs) for alternating current (AC) line-filtering performance.

## Results

### Preparation and characterization of unzipped nanostructures

**Figure 1:**
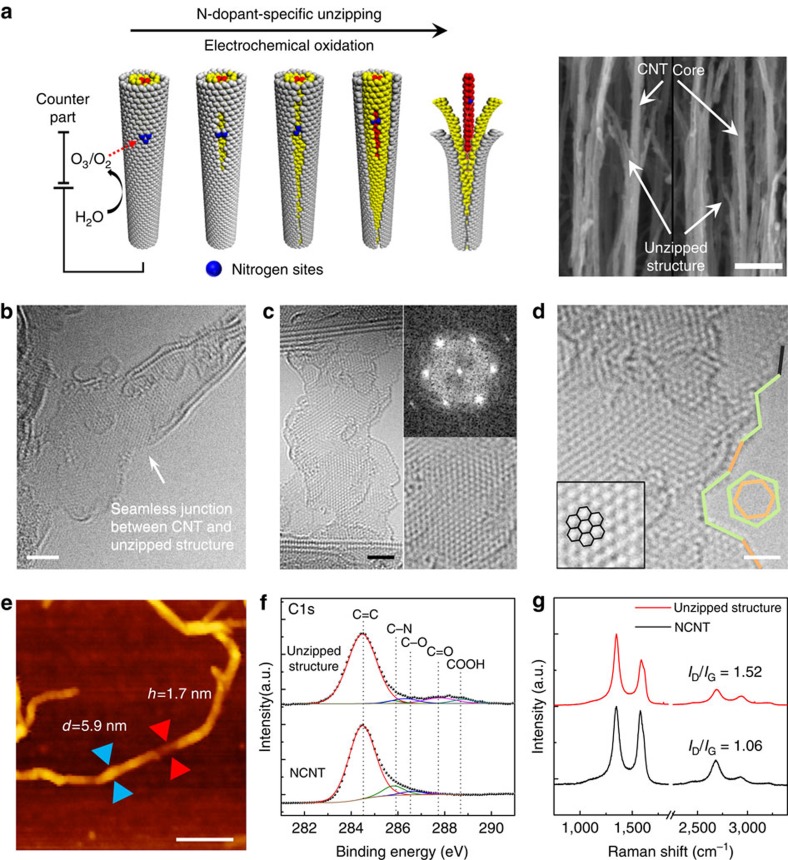
Carbon nanostructure formation by N-dopant-specific unzipping. (**a**) Schematic illustration of N-dopant-specific unzipping of NCNTs and scanning electron microscopy images of resulting unzipped nanostructures consisting of graphene nanoribbons wrapping around NCNT cores. (**b**–**d**) Aberration-corrected TEM images of unzipped nanostructures. (**b**) Seamless junction between CNT and unzipped nanoribbons. (**c**) Intact crystalline unzipped graphene nanoribbons with fast Fourier transformation analysis (top right) and magnified observation showing in-plain hexagonal carbon lattice (bottom right). (**d**) Unfurled unzipped edge with zigzag-predominant configuration. Green, orange and black colour indicate zigzag edges, armchair edges and chiral-edges, respectively. Inset shows in-plain hexagonal carbon lattice as an angle reference for edge-configuration characterization. (**e**) AFM image of unzipped nanostructure, where NCNTs and graphene nanoribbons are seamlessly connected. Blue and red arrows indicate NCNT and graphene nanoribbons, respectively. (**f**,**g**) C1s XPS and Raman spectra of unzipped nanostructures (top) and NCNTs (bottom). Scale bars are 50 nm in **a**, 2 nm in **b**,**c**, 1 nm in **d** and 100 nm in **e**.

Figure 1 presents the carbon nanostructures produced from the electrochemical unzipping of NCNT forest. Unzipping procedure is schematically described in Fig. 1a. While NCNT forest is immersed in 1 M H_2_SO_4_ aqueous solution at a positive potential of 0.6–0.8 V, electrochemically developed high-energy oxygen species react with the carbon atoms nearby the N_p_-dopant sites and initiate unzipping^24^,^28^. As unzipping propagates at outermost walls, inner walls are exposed to H_2_SO_4_ solution and subsequently unzipped (Supplementary Fig. 1a). Eventually, the carbon nanostructures, consisting of unzipped graphene nanoribbons wrapping around NCNT core (Fig. 1a and Supplementary Fig. 1b,c) are formed, as confirmed by field-emission scanning electron microscopy. Close observation of the unzipped outer NCNT walls with aberration-corrected transmission electron microscopy (TEM) reveals the seamless junction between intact sidewalls and unzipped parts (Fig. 1b).

Figure 1c shows an aberration-corrected TEM image of the unzipped outmost CNT walls. Significantly, the basal plane of the unzipped graphene sheets maintains the intact crystallinity of parent NCNTs, as characterized by fast Fourier transform analysis. The configuration of edges developed during unzipping reaction is characterized with the angle between in-plane hexagonal carbon lattice and edge direction^29^,^30^ (Fig. 1d). Zigzag edge configuration (green line) predominates in the unzipped structures (Supplementary Fig. 2). Figure 1e shows an atomic-force microscopy (AFM) image of 1.7-nm-thick tri-layer graphene sheet produced from 5.9-nm-diameter triple-walled NCNTs (Supplementary Fig. 3). The lateral width of unzipped structures is slightly larger than the diameter of parent NCNTs as expected (Supplementary Fig. 4). After unzipping, solvent exchange with volatile alcohols and subsequent critical point drying restore the vertically aligned morphology (Supplementary Fig. 5).

Chemical structure of typical unzipped materials was characterized by X-ray photoelectron spectroscopy (XPS) and Raman spectroscopy. Deconvoluted C1s XPS spectrum (Fig. 1f) shows the five distinct peaks for graphitic structure (C–C/C=C at 284.6 eV), carbon–nitrogen covalent bond (C–N at 285.7 eV), hydroxyl/epoxy groups (C–O at 286.6 eV), carbonyl group (C=O at 287.8 eV) and carboxyl group (O–C=O at 288.7 eV), respectively. Substantial decrease of the C–N peak intensity after unzipping signifies a specific chemical modification involved with N-dopant sites. Appearance of the minor peaks for oxygen functional groups demonstrates a low level of oxidation during unzipping. In the Raman spectra (Fig. 1g), a moderate increase in the G to D peak intensity ratio (*I*_D_/*I*_G_) from 1.06 to 1.52 and up-shift of G band position from 1,581 to 1,588 cm^−1^ result from the decrease of in-plane sp^2^ domain size upon C=C bond breaking reactions^26^,^31^,^32^,^33^.

### Reaction mechanism for N-dopant-specific unzipping

**Figure 2:**
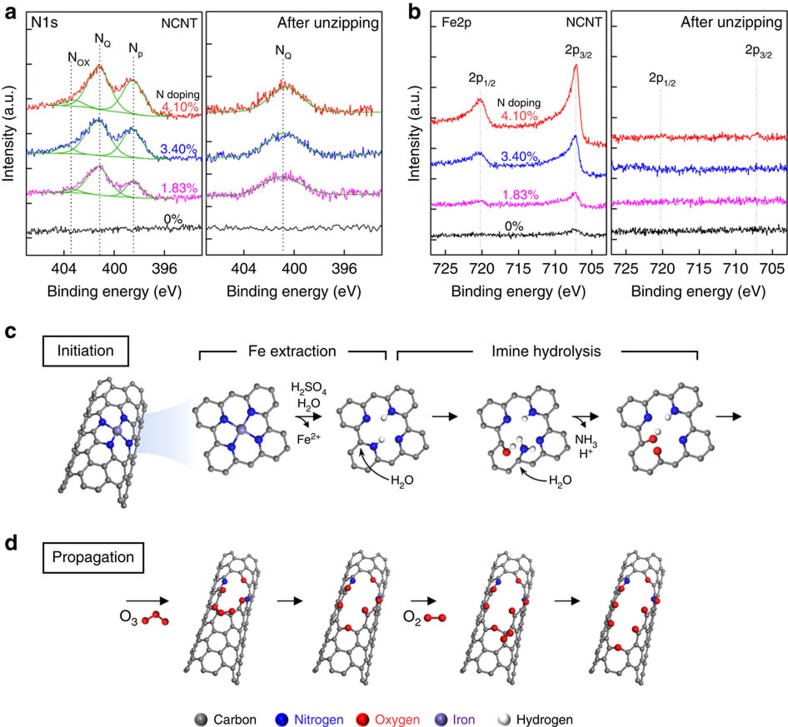
Reaction mechanism for N-dopant-specific unzipping. (**a**,**b**) N1s and Fe2p XPS spectra before and after unzipping. N_p_ (**a**) and Fe (**b**) completely disappear after unzipping. (**c**,**d**) Reaction mechanism for initiation and propagation stages. Fe^2+^ is extracted from FeN_4_ site in NCNTs under acidic positive-biased condition, and subsequent acid-catalysed imine hydrolysis undergoes (**c**). Unzipping propagates along the longitudinal direction through continuous C=C bond breaking with high-energy oxygen species, such as singlet O_2_ or O_3_ molecules (**d**).

The high-crystalline unzipping of NCNTs with low oxygen content implies a well-defined chemical reaction rather than violent random oxidative reactions. N1s XPS results of NCNTs before and after unzipping (Fig. 2a) reveal that N_p_ peak is specifically reduced during unzipping, whereas quaternary nitrogen (N_Q_) is not influenced. Concurrently, Fe2p XPS results (Fig. 2b) indicate the disappearance of Fe species in the unzipped structures. In our plasma-enhanced chemical vapour deposition growth of NCNTs from Fe catalyst, one Fe atom taken from catalyst complexes with four N_p_ atoms to generate FeN_4_ moieties in the graphene plane^34^ (Supplementary Table 1). In an acidic unzipping environment, two protons (2H^+^) can replace the Fe^2+^ ion to form H_2_N_4_ moiety (Supplementary Fig. 6 and Supplementary Note 1), which further undergoes acid-catalysed imine hydrolysis reaction (Supplementary Fig. 7). Protonation at the imine nitrogen forms iminium ion, which reacts with water followed by the proton transfer. This process could lead to the formation of carbonyl and hydroxyl groups along with the removal of NH_3_, as shown in Fig. 2c. The N_p_ is thus eliminated from the graphitic plane, as verified by N1s XPS data.

To propose the detailed propagation mechanism for unzipping, we investigated on the reactive species for C–C bond cleavage. Noteworthy, the presence of ozone molecules were detected under our electrochemical oxidative conditions (Fig. 2d and Supplementary Fig. 8). The reactive ozone species preferentially attack the alkene in the α,β-position of the carbonyl group, which is highly strained by the steric repulsion between carbonyl and hydroxyl groups. Moreover, the density functional theory (DFT) computation provides a probable reaction pathway for unzipping (Supplementary Fig. 9). It was found that the resulting ozonide formed from ozone and alkene can be dissociated into two molecules of carbonyl compounds and generate an epoxide from the next nearest strained alkene. This process can be repeated by the incorporation of singlet oxygen (Fig. 2d). This propagation mechanism explains the directional unzipping to occur via sequential generation of strained alkenes that act as the key oxidation sites due to the steric constraints imposed by the carbonyl groups (Supplementary Fig. 10). Consequently, unzipping propagates along the chemically favoured direction rather than random cutting^1^,^35^. Termination of the unzipping occurs when the inmost NCNT is exposed. Partial unzipping of the inmost NCNT, which is the final electrical pathway for electrochemical unzipping, leads to the serious degradation of electrical connectivity. Insufficient electrical potential delivery via the damaged inmost wall self-terminates the unzipping process (Supplementary Fig. 11). This rational suggestion for N_p_ site-initiated unzipping mechanism provides excellent motivation for further study to fully resolve the role of heteroatom in unzipping reaction, including other possible heteroatom dopants.

### Precise control of unzipping level

An essential requirement for the customized unzipping is the precise controllability of initiation, propagation and termination steps. In our approach, the density of N_p_-dopants (unzipping initiation sites) is easily tunable by adjusting NH_3_ gas partial pressure during the synthesis of NCNTs^36^,^37^(Supplementary Fig. 12). Electrical potential is another significant parameter for highly selective unzipping reactions^19^. Undoped CNTs are inert up to 0.8 V (Supplementary Fig. 13), above which random reaction occurs and severely damages the CNT surface^38^. By contrast, N_p_-dopant-specific unzipping is triggered at the significantly reduced potential of 0.6 V (Supplementary Fig. 14). In this work, a moderate potential of 0.8 V is determined as the optimum potential value for a highly selective unzipping from N_p_-dopant sites with manageable unzipping rate.

**Figure 3:**
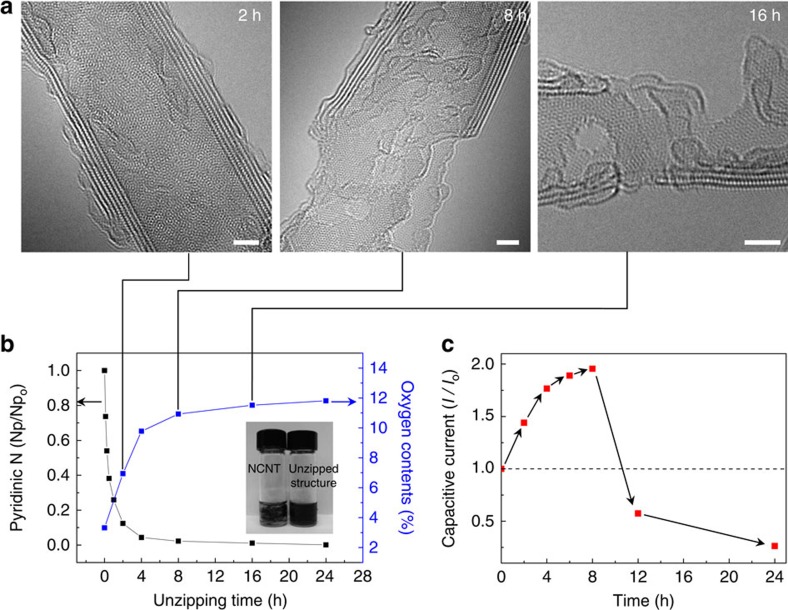
Structure and chemical evolution during N-dopant-specific unzipping of NCNTs. (**a**) Aberration-corrected TEM images of 2-, 8- and 16-h-unzipped nanostructures (in 1 M H_2_SO_4_ at 0.8 V). (**b**) N_p_ and O contents versus unzipping time. (**c**) Capacitive current in cyclic voltammogram versus unzipping time. Scale bar, 2 nm.

Control of the unzipping time (at 0.8 V) enables the fine controllability of unzipping propagation. Figure 3a shows TEM images of 2-, 8- and 16-h-unzipped structures. Although the outermost walls are selectively peeled off after 2 h, fully unzipped structures are predominantly formed after 8 h. Finally, completely torn small graphene flakes are observed after 16 h. Interestingly, hexagonal crystallinity of graphitic domains remains intact in basal plane even after 16 h. Figure 3b presents the evolution of O and N_p_ contents along with unzipping process. The drastic N_p_ content reduction at the initial stage of unzipping substantiates the selective and concurrent commencement of unzipping initiation at multiple N_p_-dopant sites, which are subsequently transformed into oxygen functional groups. The increasing rate of O or the decreasing rate of N_p_ can be correlated with the overall unzipping rate. High unzipping rate rapidly diminishes for the first 8 h and gradually reaches to near-zero in the next 16 h. The final structure obtained by prolonged 24-h-unzipping exhibits the O content of 11.9 % along with the dramatic improvement in the dispersibility of unzipped structures in polar solvents^39^ (inset optical image in Fig. 3b and Supplementary Fig. 15).

Temporal evolution of capacitive current (*I*_c_) is another measure to monitor the unzipping progress, particularly in terms of surface area enhancement^19^. *I*_c_ rapidly increases in the early stage because of the enlargement of surface area by gradual exposure of the inner surface of NCNTs, and the rate enhancement of *I*_c_ slowdowns after 4 h (Fig. 3c). The sudden reduction of *I*_c_ at 8 h reflects the serious damage of inmost wall of NCNTs that causes a loss of electrical connectivity^40^. Such a deterioration of the electrical integrity eventually leads to the self-termination of unzipping process.

### Ultrahigh-power DLCs for AC filtering

As a typical application of our unique unzipping mechanism, AC-line filtering DLC is tested. Owing to the rapid electrical response through unscathed crystalline structure and the large surface area with vertical alignment, the unzipped forest structure surpasses conventional electrode materials for AC-line filtering, including metal foil or pristine graphitic carbons.

**Figure 4:**
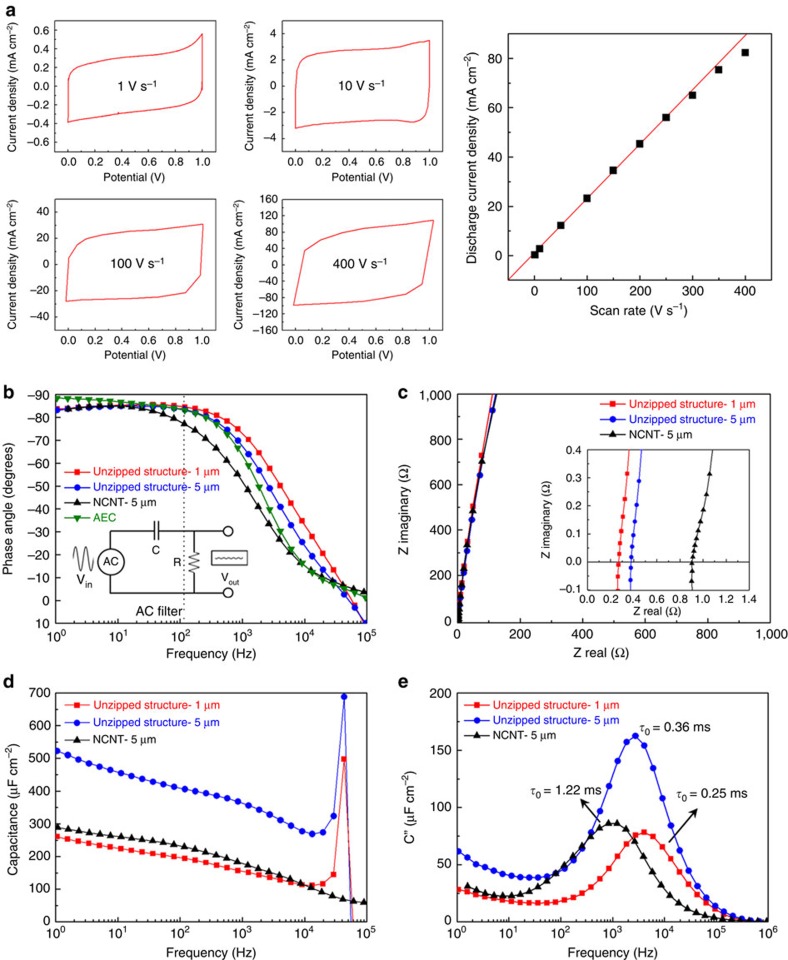
Ultrahigh-power double layer capacitors with unzipped nanostructure forest electrodes. (**a**) Cyclic voltammograms at fast scan rates. (**b**) AC impedance phase angle versus frequency; vertical dotted line indicates 120 Hz frequency. (**c**) Complex plane plot of impedance; inset shows an expanded view in high-frequency region. (**d**) Specific capacitance versus frequency assuming a series RC circuit model. (**e**) Imaginary part (C”) of specific capacitance versus frequency.

AC-line filtering DLC performance is analysed in a symmetric two-electrode configuration with identical unzipped forest electrodes over a 1-V potential window in 1 M KOH solution without any binder or conducting additive. Cyclic voltammograms (CVs) were measured at 1–400 V s^−1^ (Fig. 4a). Reproducible rectangular shape curves were obtained even at 400 V s^−1^, indicating a near-ideal capacitive behaviour with stable electrical double layer formation and fast charge propagation throughout the electrodes. The ultrahigh-power performance is also illustrated by the linear dependence of discharge current upon scan rate as well as the little change in the areal capacitance as a function of scan rate (Supplementary Fig. 16a). This fast energy delivery capability was stably maintained up to 10,000 cycles, showing 96.2 % of capacitance retention (Supplementary Fig. 16b)

Electrochemical impedance spectroscopy further confirms the outstanding power performance of the DLCs with unzipped structures. Figure 4b compares the impedance phase angles of DLCs at different frequencies for 1-μm- and 5-μm-height unzipped forests, 5-μm-height pristine NCNT forest and typical aluminium electrolytic capacitor (AEC, 10 V/220 μF). All tested DLCs show typical capacitive behaviours (near −90° phase angle) at 20 Hz but gradually evolve into resistive behaviours at higher frequencies. The DLCs made from 1-μm- and 5-μm-height unzipped nanostructures reach −45° phase angle at 8.15 and 5.32 kHz, respectively. At 120 Hz, a standard frequency for AC filter, the DLC with 1-μm-unzipped structures shows approximately −85° of the impedance phase angle, which is comparable to that of AEC or other record-high-performance DLCs^41^,^42^,^43^,^44^,^45^,^46^. The DLC with 5-μm-unzipped structures shows a slightly decreased impedance phase angle of −78° because of the elongated ion diffusion length and increased electrical resistance. The near-vertical line characteristics without semicircle and the low equivalent series resistance values from the Nyquist plots in Fig. 4c indicate that the unzipped nanostructure facilitates fast ion diffusion during charge/discharge operation.

In a DLC, series resistance, which hampers the power capability of the devices, can be simply considered as the sum of electronic and ionic resistance. The vertically aligned unzipped morphology of our electrode materials can effectively minimize the resistive elements. The vertical alignment offers idealized straight pathway for facile ion transport and homogeneous distribution of stored charges at electrode surfaces^46^,^47^. The intimate connection of the individual unzipped nanostructure to bottom current collector minimizes electronic resistance throughout the electrodes consisting of tightly attached CNT cores and unzipped nanostructures. Simultaneously, the exposure of inner CNT walls to the electrolyte and the improved wettability by hydrophilic oxygen-functionalized edges at unzipped structure allow for a greatly improved ion-accessibility to the electrode surfaces as well as the enhancement of accessible surface area^44^,^48^.

Specific areal capacitance (*C*_A_, F cm^−2^) is also calculated (equation (1))^49^. Owing to enlarged surface area, 5-μm-unzipped structure-based DLC exhibits 402 μF cm^−2^ at 120 Hz, far higher than 230 μF cm^−2^ of the 5-μm-NCNT-DLC (Fig. 4d). Notably, 1-μm-unzipped structure-based DLC demonstrates 194 μF cm^−2^ at 120 Hz, which is ∼84 % of 5-μm-NCNT-DLC, and eventually outperform at a high frequency over 9 kHz. RC time constants (*τ*_RC_, R and C are total resistance and capacitance of devices, respectively) of DLCs fabricated with 5-μm-NCNT forest, 5- and 1-μm-unzipped structures are 616, 468 and 212 μs with 2.68, 1.24 and 1.15 Ω of total resistance at 120 Hz, respectively. These values are sufficiently smaller than the standard 8.3 ms period, which is crucial for the effective AC line-filtering at 120 Hz. The unzipped nanostructures also demonstrate a very small relaxation time constant (*τ*_0_, 0.25 ms for 1-μm-unzipped structure, Fig. 4e) with moderate areal capacitance value, where *τ*_0_ is defined as the minimum time to discharge all the energy from the device with an efficiency greater than 50 % (ref. 50). In the cases of DLCs with 5-μm-unzipped structure and 5-μm-NCNT, *τ*_0_ increases to 0.36 and 1.22 ms, respectively. These represent that the DLCs constructed with the customized unzipped structures show not only ultrahigh-power performance, but also larger energy capacity at 120 Hz. This finding illustrates that the unzipped nanostructures possess enormous potential for the rapid delivery of ultrahigh power.

## Discussion

Unprecedented control of the graphene unzipping reaction is attained by substitutional heteroatom dopants. Highly specific unzipping initiation at N_p_-dopant sites is confirmed by the significantly reduced electrochemical potential for unzipping and the distinctive correlation between unzipped morphology and N-dopant density. It is noteworthy that N-dopants have been suggested as readily oxidizable sites for the conventional chemical unzipping of CNTs^51^. Our work first reports a novel N-dopant-specific unzipping mechanism based on the systematic experimental characterization and theoretical analyses. Significantly, atomic-scale double-aberration corrected TEM analysis confirms that this dopant-specific unzipping generates the graphene-based nanostructures with intact crystallinity and well-defined atomic configuration at unzipped edges. These unique features are feasible not only for multi-walled CNTs, but also for single-walled CNTs while maintaining the electrical connectivity through partially unzipped structures (Supplementary Fig. 17). Our novel reaction mechanism exploiting heteroatom dopants is clearly distinguishable from other preexisting unzipping methods, and addresses the key challenges, including the precise controllability of unzipping reaction and the preservation of intact crystalline graphene basal plane during unzipping process.

In this work, we report the ultrahigh-power performance of unzipped structure-based DLCs. In contrast to the intensive research efforts for high-energy-density supercapacitors^15^,^52^,^53^,^54^,^55^, power performance (or frequency response) enhancement of supercapacitors has attracted less research attention. Recently, ultrahigh-power supercapacitors capable of operating above 120 Hz have been newly recognized because of their possibility to replace the conventional AECs in AC filter circuit^46^. AECs are among the largest components in electronic circuit system that impede the reduction of circuit size, principally due to their low specific energy densities^55^. Supercapacitors with frequency response comparable to that of AECs are highly demanded for the effective miniaturization of electronic circuits and relevant portable devices. Our DLC with 1-μm-height unzipped structure exhibits rectangular shape CV curves even at the fast scan rate of 400 V s^−1^, −85° of the impedance phase angle, 212 μs of *τ*_RC_ and 0.25 ms of *τ*_0_ at 120 Hz. As summarized in Table 1, this remarkable power performance and AC frequency response are comparable or even superior to those of conventional AECs or the prime tier carbon nanomaterial-based devices reported thus far. Notably, the most proximate value to −90° in impedance phase angle and the lowest level of resistance (1.15 Ω) at 120 Hz indicate that the DLC with 1-μm-unzipped structure can properly function as well as typical AEC at 120 Hz. Besides, our unzipped nanostructure electrode presents a volumetric capacitance (*C*_V_=0.95 F cm^−3^) at 120 Hz, significantly higher than previous carbon based-electrodes. This *C*_V_ value is approximately three orders of magnitude higher than those of typical AECs (∼10^−4^ F cm^−3^)^43^,^44^,^56^.

As demonstrated from the record-high power DLCs, delicate manipulation of graphene-based nanostructures may offer straightforward routes to high-performance applications. The highly specific reaction features of our unzipping mechanism can serve as a generic principle to diversify graphene-based nanostructures, whose physicochemical properties are strongly dependent on their structural dimension and defect formation^1^,^2^,^3^,^4^,^5^,^6^,^7^,^8^,^9^. In principle, controlled cutting of sp^2^-hybridized carbon plane allows the transformation of structural dimension among zero-, one-, two- and three-dimensions. Overall, customized dopant-specific unzipping of graphene-based materials possibly guides us to rarely explored opportunity for multi-dimensional material design with atomic level precision as well as physicochemical property tuning, which are generally desired for energy storage, environmental remediation, nanomedicines and other versatile applications. Moreover, this precisely controllable unzipping mechanism grants a significant step forward to idealized intact crystalline graphene nanostructures in conjunction with the potential advances in the precise control of dopant position, dopant density and the chirality of CNTs.

## Methods

### Materials synthesis

NCNTs were grown by plasma-enhanced chemical vapour deposition with Fe catalyst. The density of N dopants was easily controllable by adjusting NH_3_ gas partial pressure and plasma power (Supplementary Fig. 12). As-grown vertical NCNT forest on SiO_2_/Si was floated on diluted hydrofluoric acid (HF) solution to separate NCNT forests from SiO_2_/Si substrate by wet-etching of SiO_2_ layer. The floated NCNT forests were carefully transferred onto a glassy carbon electrode. To enhance the mechanical and electrical contact between NCNT forests and glassy carbon electrode, the recovered electrode was thermally annealed at 65 °C for 12 h. The NCNT electrode was used as working electrode in typical three electrode configuration with mercury/mercury (I) sulfate reference electrode and platinum wire (counter electrode). The unzipping was carried out at room temperature in 1 M H_2_SO_4_ solution with positive electrical potential from 0.5 to 1.0 V for various unzipping period. Before each unzipping reaction, the electrolyte was purged with high purity N_2_ gas over 10 min.

### Materials characterization

HR-TEM images were collected on a double-aberration corrected HR-TEM (Titan G2 60–300, FEI) located at KAIST Analysis Center for Research Advancement, operating with a monochromator (excitation 0.06) at 80 kV accelerating voltage. The structural changes were also measured by field-emission scanning electron microscope (Magellan400, FEI). Samples were prepared by critical point drying to maintain the as-synthesized vertically aligned structure. AFM images were obtained with scanning probe microscope (MultiMode 8, Bruker). Samples were prepared by drop-casting from ethanol suspensions onto SiO_2_/Si substrate. Raman and XPS spectra were recorded with ARAMIS (Horiba Jobin Yvon) and Sigma probe (Thermo VG Scienfitic), respectively.

### Electrochemical characterization

Unzipped nanostructures (1 cm^−2^) on Pt foil were used as an electrode for ultrahigh-power DLCs. Before DLC assembly, the unzipped structures were electrochemically reduced at −0.6 V for 30 min to remove dispensable oxygen functional groups. The prototype DLCs were composed of two identical unzipped nanostructure electrodes and separator. All electrochemical characterization was carried out with a potentiostat (Bio-Logic, SP-200) using 1 M KOH solution as an electrolyte under ambient environment. All CVs in Fig. 4 were collected in 10th cycle at each scan rate. Electrochemical impedance spectroscopy measurements were conducted with sinusoidal signal of 10 mV amplitude at various frequencies ranging from 10 mHz to 1 MHz. A series-RC circuit model (equation (1)) was used to calculate the frequency response-specific areal capacitances (*C*_A_, F cm^-2^)


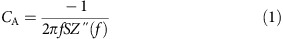


where *f* is frequency; *S* is the total surface area of active materials region on both planar electrodes (cm^2^); *Z*"(*f*) is the imaginary part of the impedance.

The frequency specific volumetric capacitances (*C*_V_, F cm^−3^) was calculated with equation (2)


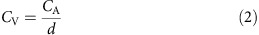


where *C*_A_ is the specific areal capacitance calculated by equation (1); *d* is total thickness of active material.

The specific capacitance of DLCs prepared with unzipped nanostructures could be calculated by using *C*′(*f*) and *C*"(*f*),


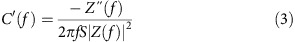



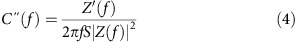


where *C*′(*f*) is the real part of specific capacitance; *C*"(*f)* is the imaginary part of specific capacitance; *Z*′(*f*) and *Z*"(*f*) are real and imaginary parts of the impedance; |*Z*(*f*)| is the absolute value of the impedance.

### DFT calculation for unzipping mechanism

To understand the mechanism for electrochemical unzipping of CNTs, first-principles DFT calculation was performed using Vienna *Ab-initio* Simulation Package codes. The Perdew–Burke–Emzerhof exchange-correlation energy functional and projected-augmented wave potentials were employed. A plane-wave kinetic energy cutoff of 400 eV and 1 × 1 × 1 gamma k-point were used for 12 unit cells of (5, 5) single-walled CNT with 240 atoms. The supercell size is 20 × 29.514 × 20 Å^3^ with the tube axis along the *y* direction, and the diameter of model is about 6.726 Å. Atomic forces were relaxed <0.025 eV Å^−1^.

## Additional information

**How to cite this article:** Lim, J. *et al*. Dopant-specific unzipping of carbon nanotubes for intact crystalline graphene nanostructures. *Nat. Commun.* 7:10364 doi: 10.1038/ncomms10364 (2016).

## Supplementary Material

Supplementary Information Supplementary Figures 1-17, Supplementary Table 1, Supplementary Note 1 and Supplementary References.

## Figures and Tables

**Table 1:** A comparison of electrochemical performance of various carbon-based DLCs.

**Device**	**Reference**	**Materials**	**Phase angle at 120 Hz (°)**	***τ***_**RC**_ **at 120 Hz (ms)**	**Resistance at 120 Hz (Ω)**	***τ***_**0**_**(ms)**	***C***_**A**_ **at 120 Hz (mF cm**^−2^**)**	***C***_**V**_ **at 120 Hz (F cm**^−3^**)**
Sandwich-type device	This work	1** **μm-Unzipped nanostructure	85	0.21	1.15	0.25	0.19	0.95*
	Miller *et al*.^46^	VG	82	∼ 0.2	1.1	NA	0.09	0.73*
	Sheng *et al*.^45^	ErGO	84	1.35	3.4	0.24	0.28	0.28*
	Du and Pan^57^	CNTs	< 75	NA	NA	1.5	NA	NA
	T. Nathan-Walleser^58^	TrGO	30	2.3	NA	4.1	3.6	1.8*
	Pech *et al*.^50^	AC	∼1	NA	NA	700	NA	NA
	El-kady *et al*.^43^	AEC (63 V/220 μF)	NA	NA	NA	NA	NA	∼10^−4^
	Lin *et al*.^44^	AEC	83.9	0.14	NA	NA	NA	NA
Micro-device	Lin *et al*.^44^	G/CNTCs	81.5	0.20	21.3	0.82	0.23	0.23*
	El-kady *et al*.^43^	LSG	< 30	NA	NA	> 1.6	NA	NA
	Pech *et al*.^50^	OLC	NA	NA	NA	26	NA	NA

AC, activated carbon; AEC, aluminium electrolytic capacitors; CNT, carbon nanotube; DLC, double-layer capacitor; ErGO, electrochemical reduced graphene oxide; G/CNTC, graphene-CNTs carpet; LSG: laser-scribed graphene; NA, not applicable; OLC, onion-like carbon; TrGO, thermally reduced graphene oxide; VG, vertical graphene.

All of the data are the best result in impedance phase angle in each literature.

^*^The values are estimated from the value of *C*_A_ at 120 Hz and the thickness of only active material in each literature (equation (2)).
