# Supplementary material for: Dopant-specific unzipping of carbon nanotubes for intact crystalline graphene nanostructures
Source: Nat Commun. 2016 Jan 22;7:10364. doi: 10.1038/ncomms10364 (PMC4735752; doi:10.1038/ncomms10364)
Supplement: Supplementary Information — Supplementary Figures 1-17, Supplementary Table 1, Supplementary Note 1 and Supplementary References. [file ncomms10364-s1.pdf]

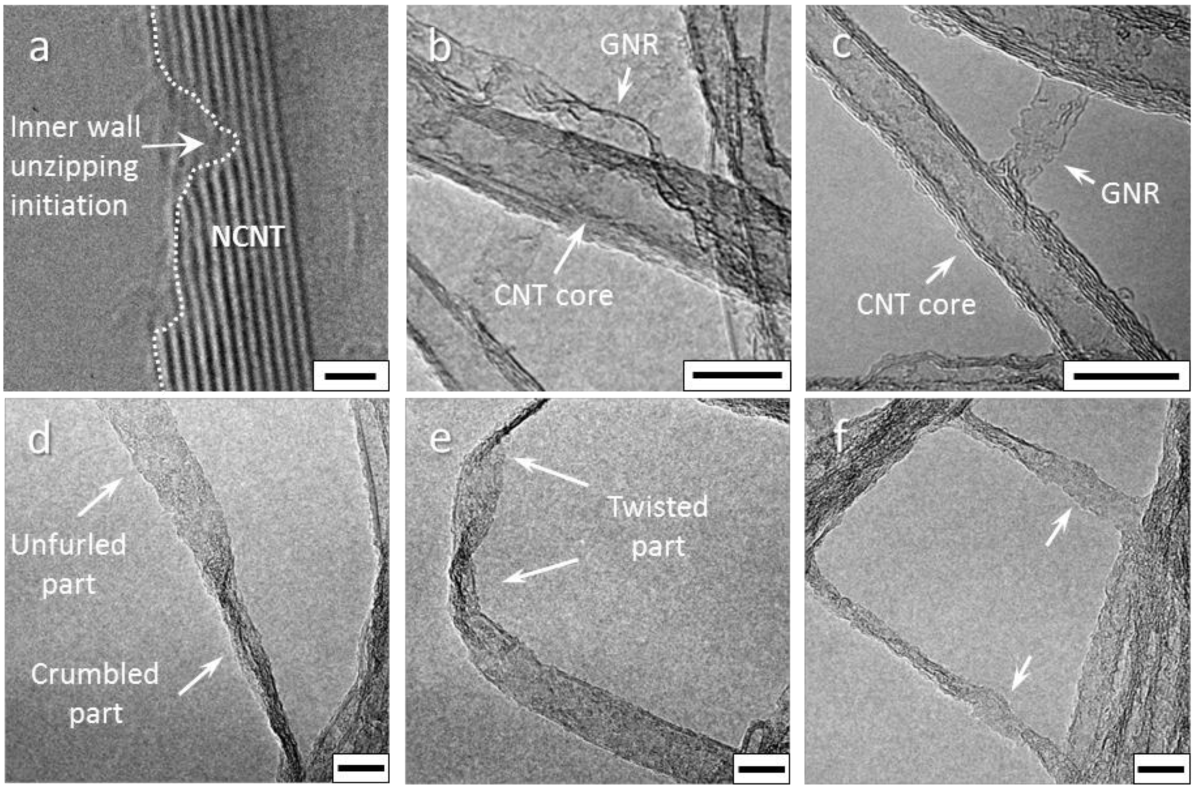

Supplementary Figure 1. HR-TEM images of unzipped carbon nanostructures by N-dopant-specific unzipping of NCNTs.

a, Sequential unzipping of inner wall of NCNTs **b,c,** Unzipped nanostructures consisting of graphene nanoribbon (GNR) wrapping around CNT core. **d-f**, various structures of unzipped nanostructure. Scale bar is 2 nm (a) and 10 nm (b-g).

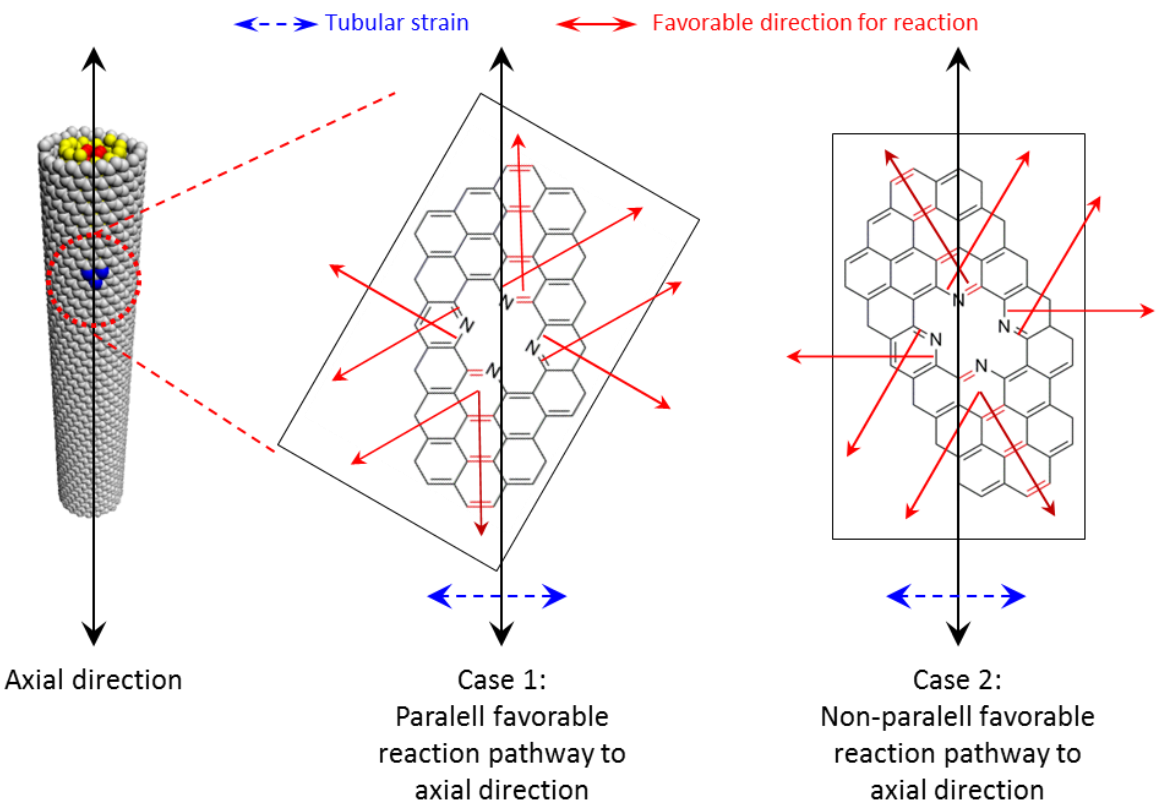

Supplementary Figure 2. Influence of CNT chirality on the edge-configuration of unzipped graphene structures.
In principle, the CNT unzipping mechanism is driven by the competition of tubular strain (blue arrow) and chemically favorable reaction direction[1] (red arrow). In the unzipping of an armchair nanotubes (chiral vector is (n, n), case 1), these two factors are completely constructive such that unzipping principally propagates along the tube axial direction, developing zigzag edge-configuration. By contrast, in the case of a zigzag or chiral nanotube (chiral vector (n, 0) or (n, m), case 2), there is a competition between these two factors and the unzipping propagate in instantaneous balance of those, generating zigzag- and armchair-mixed edge configuration. In our work, the chirality of NCNTs is not controlled. Therefore, despite of the proposed selective zigzag-edge-developing unzipping mechanism by reactive oxygen species, zigzag, armchair, and chiral edge configurations coexist in the real unzipped structures, as shown in Figure 1d. It is rationally explained with the presence of the induced mechanical stretching from tubular structure and the chirality of CNTs, which can significantly influence the resulting edge shape, as estimated by R. P. B. dos Santos et al.[2]. Nonetheless, zigzag edge configuration is still predominant in Figure 1d, as the main driving factor for unzipping process is the chemical C=C bond cleavage, as rationally discussed in the reaction mechanism section (Figure 2).

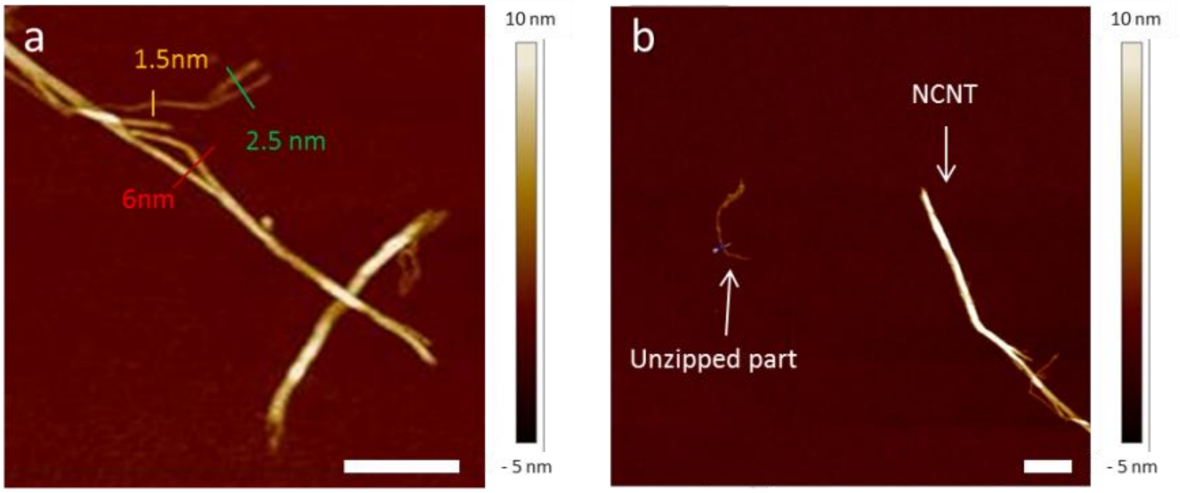

Supplementary Figure 3. AFM images of unzipped nanostructures produced from N-dopant-specific unzipping process.
The resultant nanostructures show various layer thickness depending on the original NCNT wall number. Scale bar is 300 nm.

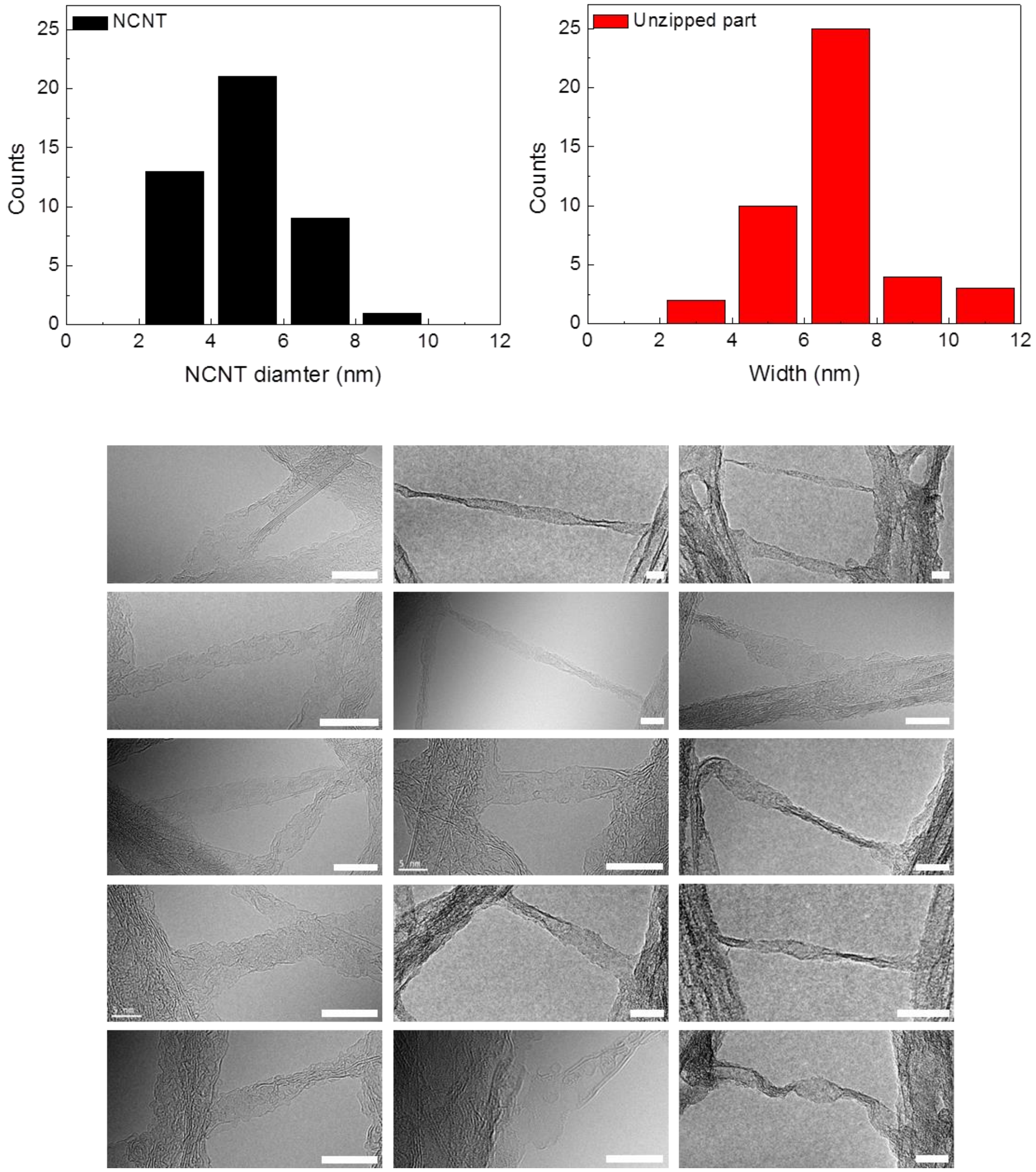

Supplementary Figure 4. The CNT diameter distribution before and after unzipping. The diameter of CNTs and the width of fully unzipped parts were measured to investigate the statistics chart (up). TEM images show fully unzipped part in the unzipped nanostructure (bottom). Scale bar is 10 nm.

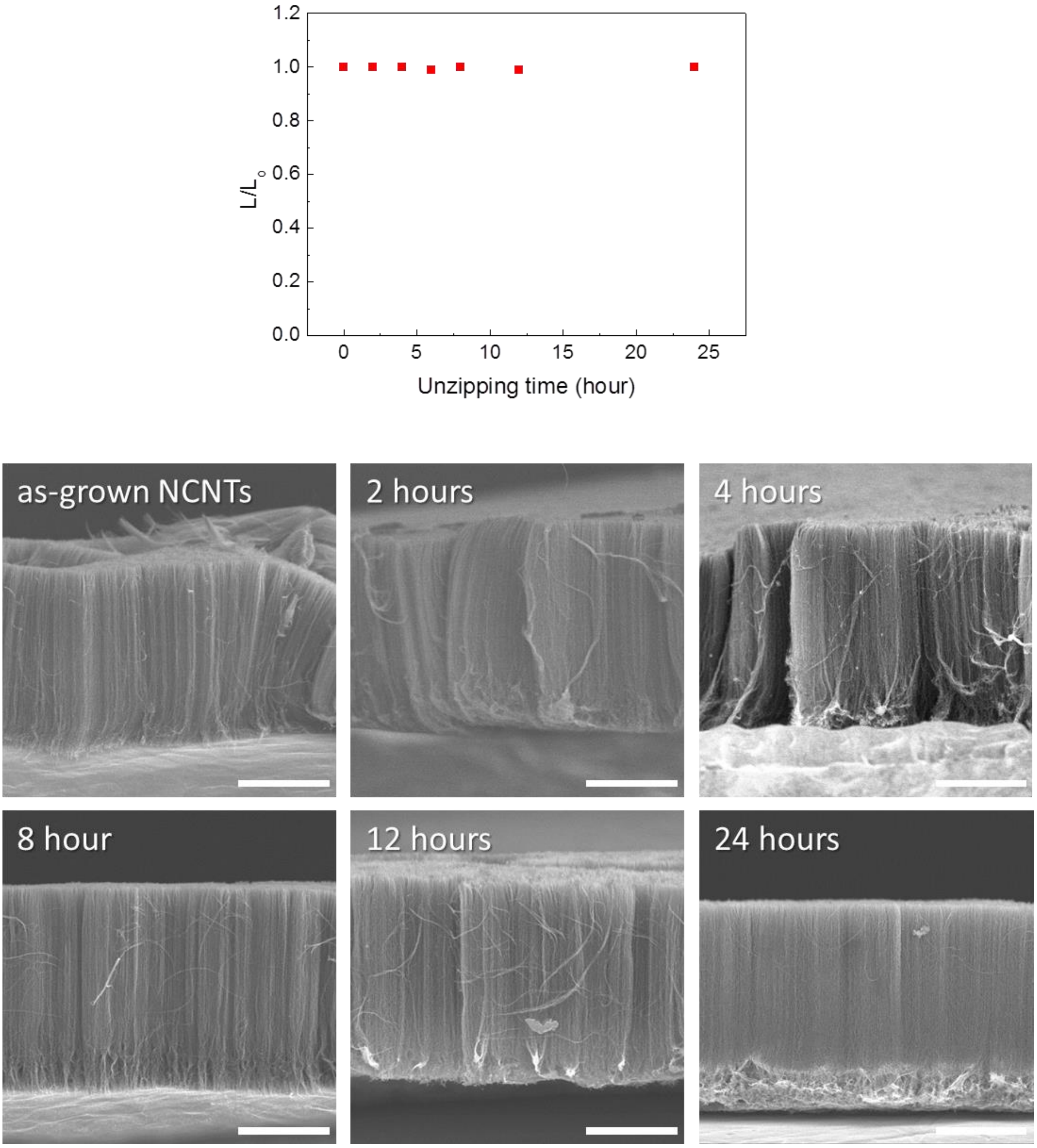

Supplementary Figure 5. SEM images of vertical unzipped nanostructure forest and the change in the length of CNTs after unzipping process.

The influence of unzipping time on the length of the unzipped nanostructures was investigated by SEM measurement. SEM images show vertically aligned NCNTs and the resulting unzipped nanostructure for various unzipping time. It shows that the unzipping process doesn't cause noticeable change in the length of the unzipped structures. This is attributed to the longitudinal unzipping of carbon nanotubes. Scale bar is 10 μm.

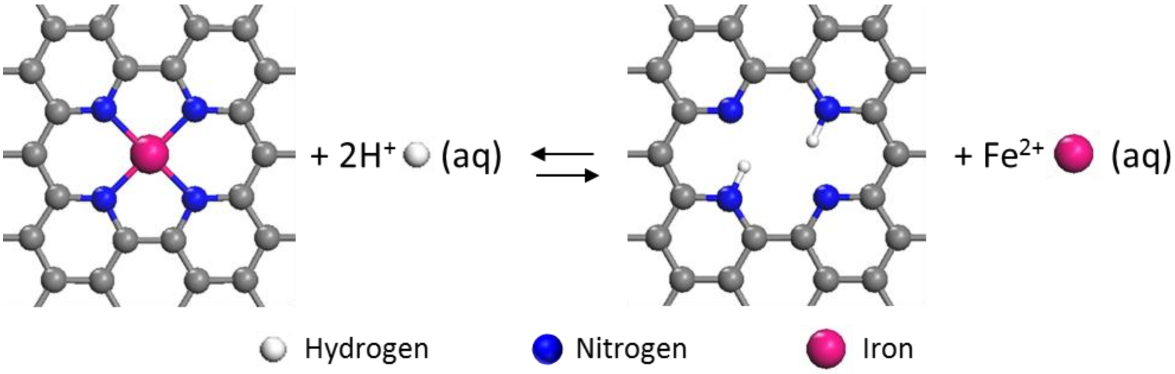

Supplementary Figure 6. Fe extraction from FeN_4 site in NCNTs under an acidic unzipping environment. See Supplementary Note 1 for further discussion.

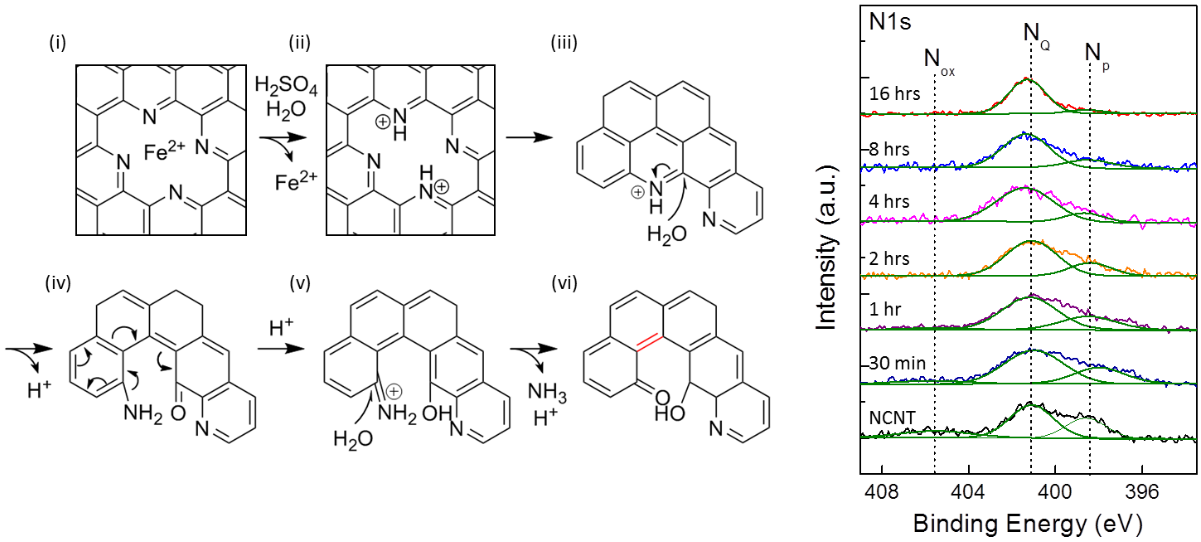

Supplementary Figure 7. Acid-catalyzed imine hydrolysis in unzipping process
Temporal analysis of N1s XPS spectra clearly shows that prydinic N (N_p) is strongly involved in the unzipping process. While the peak intensity for quaternary N (N_Q) is maintained, the peak intensity for N_p drastically decreases from the early stage of unzipping. In our unzipping condition, acid-catalyzed imine hydrolysis reaction, which is the crucial unzipping initiation step, can be accelerated. The proton (H^+) generated by water electrolysis increases the concentration of H^+ around NCNTs, which plays a role as catalyst for acid-catalyzed imine hydrolysis[3]. Additionally, the positive potential applied to the anode activates NCNTs to be more reactive for 1, 2-addition of water by extracting electrons from NCNTs. Consequently, the initiation reaction can be promoted. Detailed scheme for unzipping initiation step, consisting of Fe extraction (i-ii) and acid-catalyzed imine hydrolysis (iii-vi), and the temporal change of N1s XPS spectra during unzipping reaction.

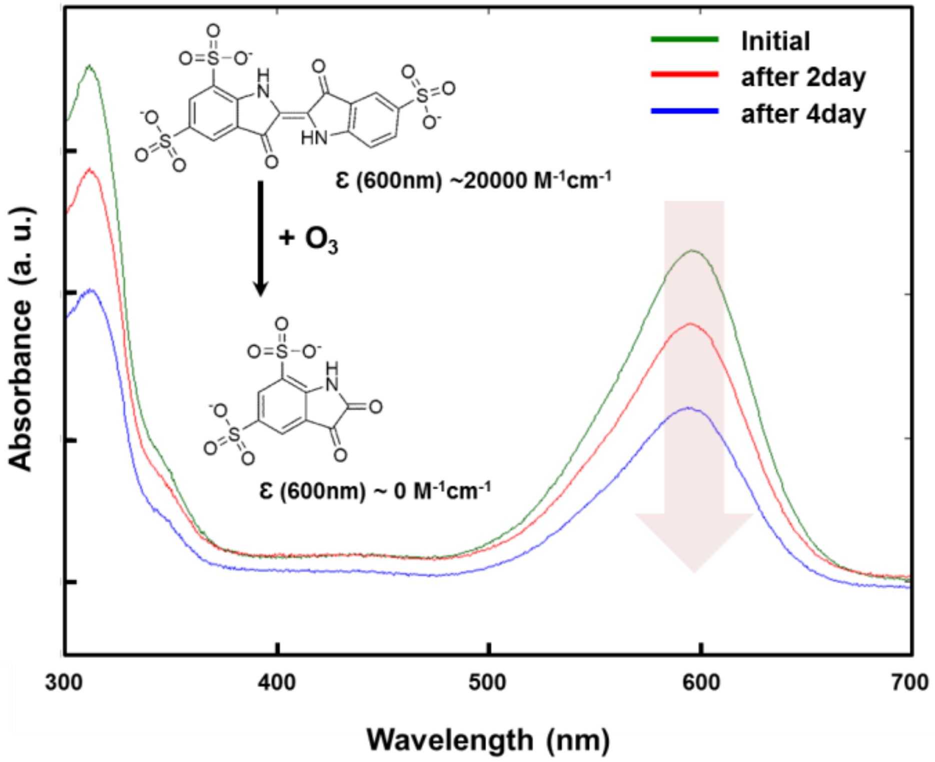

Supplementary Figure 8. Generation and detection of high-energy oxygen species
High-energy oxygen species (singlet oxygen, ozone, etc.), which breaks C=C bonding of graphitic wall of NCNTs, were detected by checking UV-VIS spectra of potassium indigotrisulfonate. This chemical has been widely used for detection of reactive oxygen species, such as ozone. Potassium indigotrisulfonate solution of certain molar concentration was dropped into 1 M H_2SO_4 and unzipping process was carried out at 0.8 V (vs. MMS). As unzipping process proceeded, the major peak of Potassium indigotrisulfonate at 600 nm gradually deceased. This confirms the existence of high-energy oxygen species generated from water-electrolysis under unzipping condition.

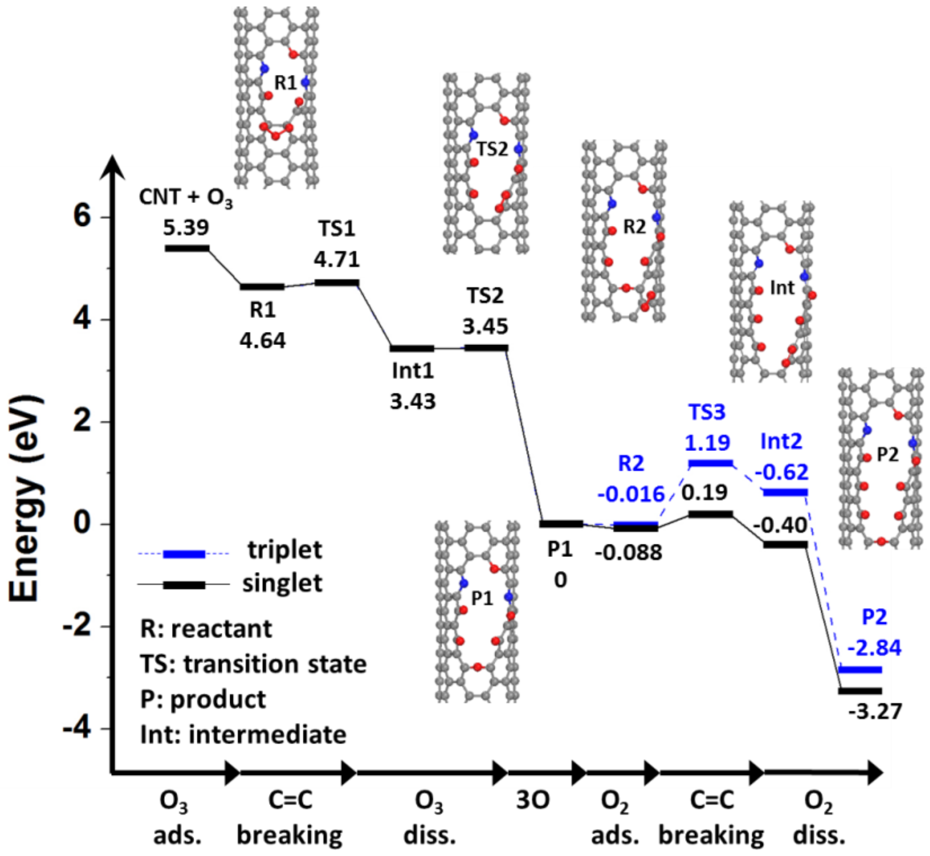

Supplementary Figure 9. Proposed propagation reaction pathway

Supplementary Fig. 8 shows the relative energy diagram along the reaction pathway, and more detailed configurations for each phase. The notations of R, TS, P, and Int indicate reactant, transition state, product, and intermediate, respectively. The first step is the adsorption of O_3 with the adsorption energy of 0.75 eV. The adsorbed O_3 forms pentagon with two carbons at the pore along the tube axis (R1), and subsequent C-C bond breaking and the dissociation of O_3 to O_2 and O occurs with a very small energy (0.07 eV, TS2). The resultant O_2 is dissociated into 2O, the one of which transfers to next C=C bond to form epoxide ring shown in P1. Subsequent unzipping reaction may proceed with O_2. After O_2 adsorption at one side of the epoxide ring (R2), the O_2 is decomposed by a similar reaction process with TS2 along with the formation of epoxide ring (Int), and finally yield 5O structure (P2). By repeating the C=C cleavage by O_2, fully unzipped configuration can be generated. Interestingly, meta-stable singlet O_2 (black line) is considered to be a reasonable unzipping propagation species comparing to triplet O_2 (blue line) with relatively higher energy barrier ($E_{ads,\ triplet}$=0.016eV, $E_{ads,\ singlet}$=0.088eV, $E_{a,\ triplet}$=1.21eV, $E_{a,\ singlet}$=0.28eV).

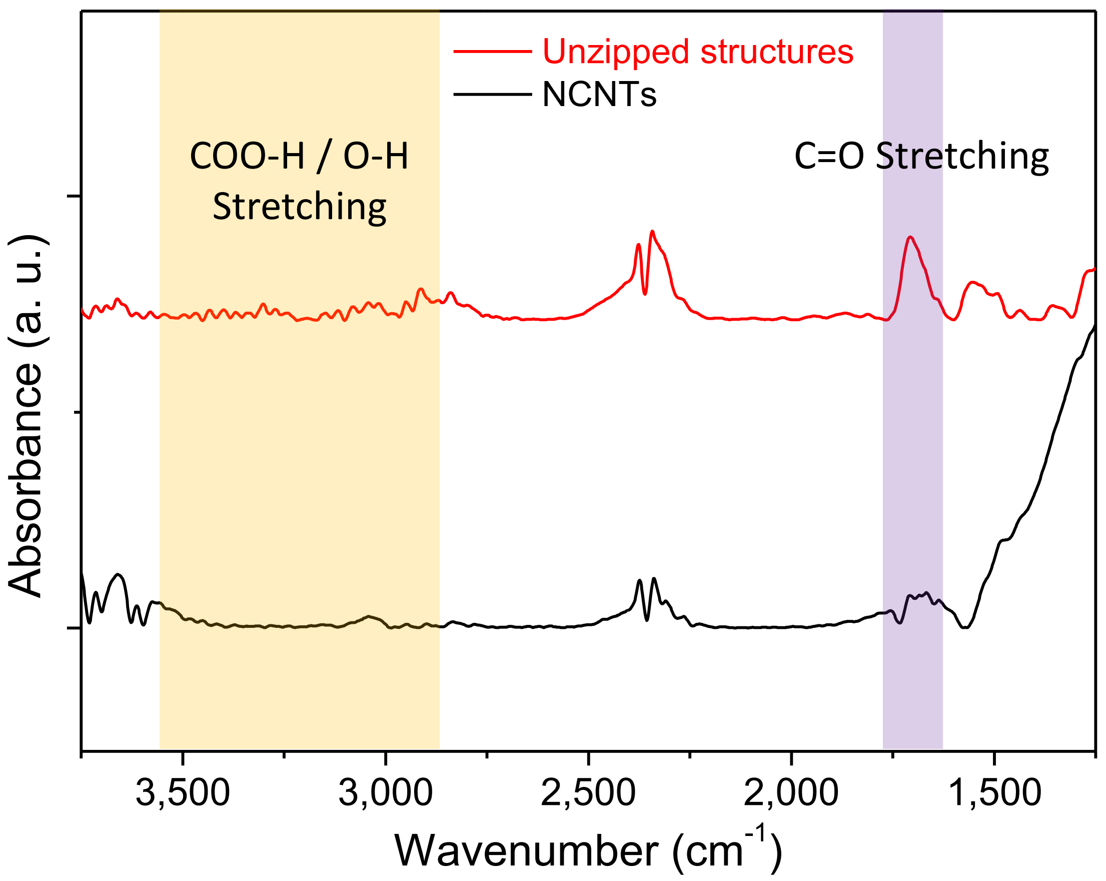

Supplementary Figure 10. ATR–IR spectroscopy of unzipped nanostructures and NCNTs.

Attenuated-total reflection infrared (ATR–IR) spectroscopy was carried out for the unzipped nanostructures, which are produced by 8-hours-unzipping at 0.8 V in 1M H_2SO_4, to characterize the formation of surface functional groups. C=O stretch (1,707 cm^{-1}) and the negligible appearance of COO-H / O-H stretch (~3,600 ~ 2,800 cm^{-1}) were detected. This result confirms that our unzipping method principally develops C=O bond at unzipped edges, which is consistent with our proposal for unzipping reaction mechanism.

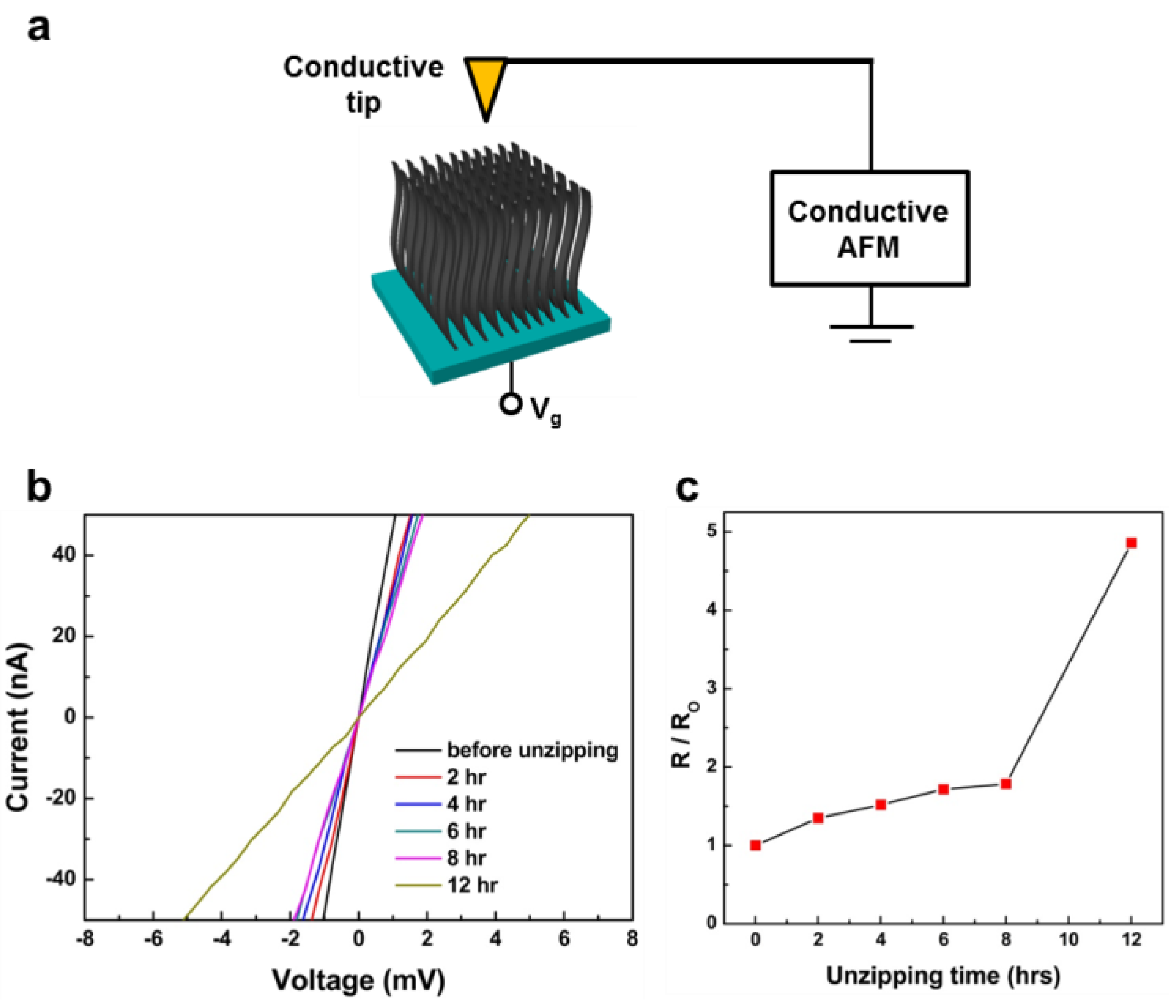

Supplementary Figure 11. I-V measurements with unzipping time.
a, Schematic for experimental set up. **b, c** I-V curves and calculated resistances with unzipping time. To investigate the decrease of electrical conductivity of NCNTs during unzipping process, we monitored the change of resistance using conductive AFM. As-grown NCNT forests were transferred onto platinum foil. Unzipping was conducted under optimized condition (in 1M H_2SO_4, at 0.8 V vs. MMS, for 8 hours). After predetermined periods of unzipping, small pieces of unzipped sample were collected while other part undergoes further unzipping. The collected sample was thoroughly washed with D.I water. After solvent exchange with ethanol, critical point drying process was employed to obtain vertically aligned graphene nanostructures for resistance measurement. As shown in Supplementary Fig. 11b, the resistance (the slope of I-V curve) abruptly increases between 8 and 12 hours of unzipping period. Supplementary Fig. 11c clearly shows the increase of resistance of NCNTs during unzipping.

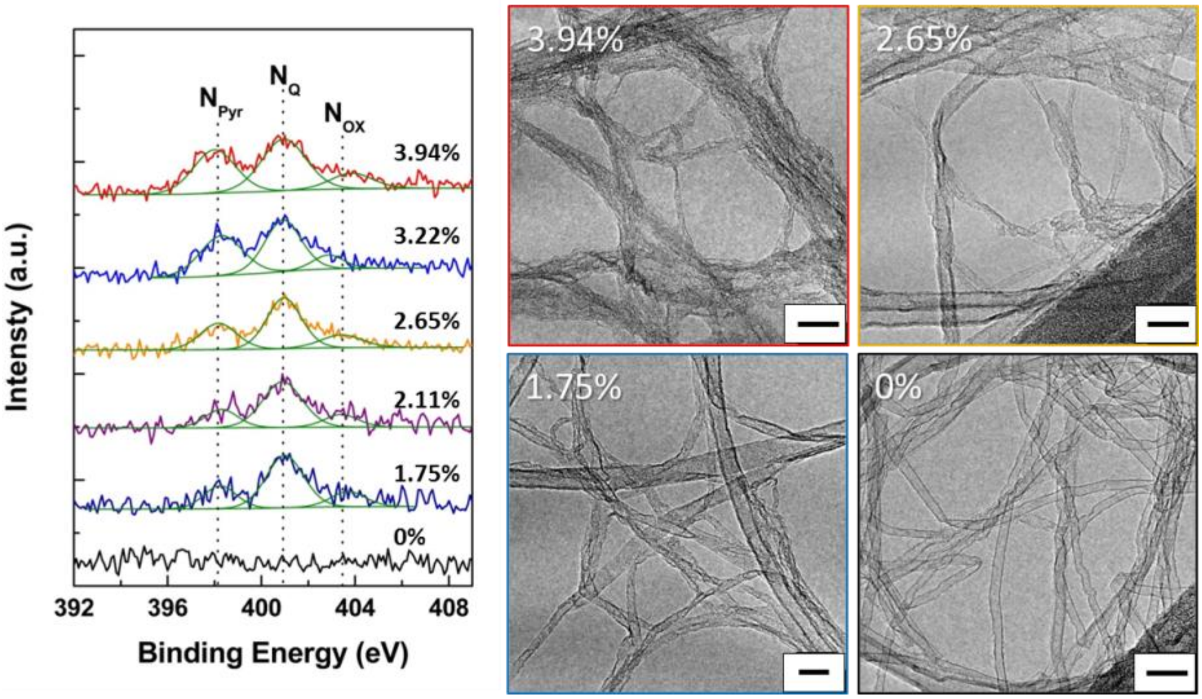

Supplementary Figure 12. TEM images of unzipped nanostructures produced from NCNTs with different N-doping levels.

The density of N_p site in NCNTs is easily manipulated by adjusting NH_3 partial pressure and plasma power during NCNT growth by PECVD. To investigate the correlation between the density of N_p-sites and unzipping reaction initiation, we synthesized NCNTs with various nitrogen (N) doping contents (atomic ratio) of 0, 1.75, 2.65, 3.94 % and conducted unzipping process in 1 M H_2SO_4 solution for 8 hours at 0.8 V (vs. MMS). As shown in TEM images, unzipping level is clearly correlated with N-dopant content. Scale bar is 20 nm.

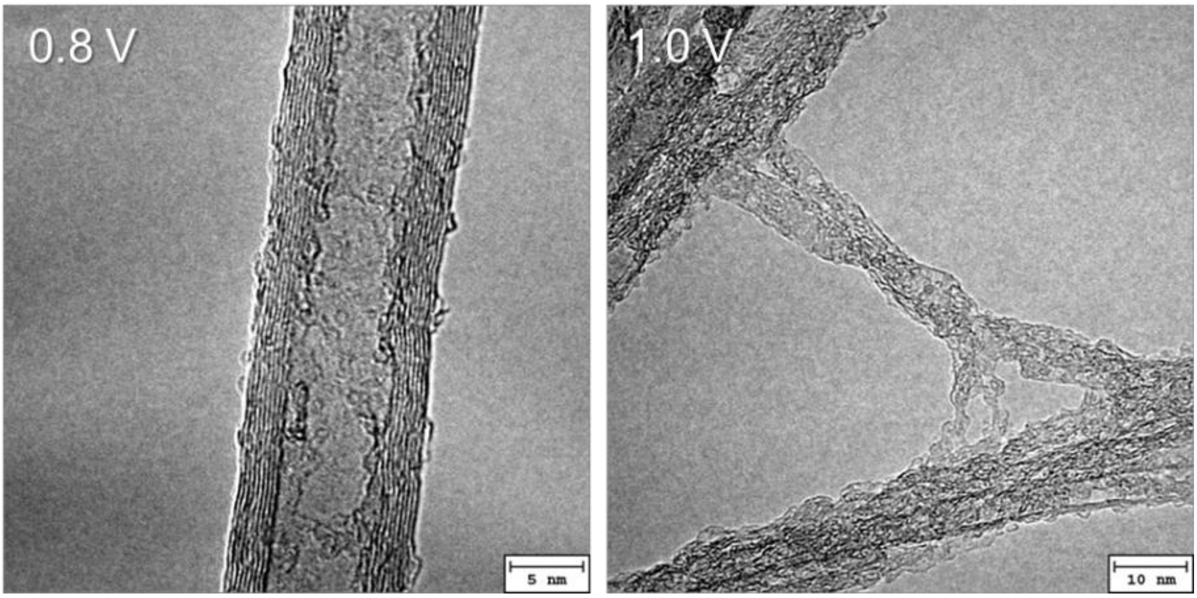

Supplementary Figure 13. TEM images of pristine undoped CNTs after electrochemical unzipping at 0.8 V and 1.0 V.

Electrochemical unzipping process was carried out with pristine CNTs under same experimental condition with NCNTs case (in 1M H_2SO_4, at 0.8 V vs. MMS, for 8 hours). Unlike NCNTs, unzipping was not observed by TEM analysis. By contrast, at 1.0 V of electrical potential, pristine CNTs was completely and randomly damaged and torn up.

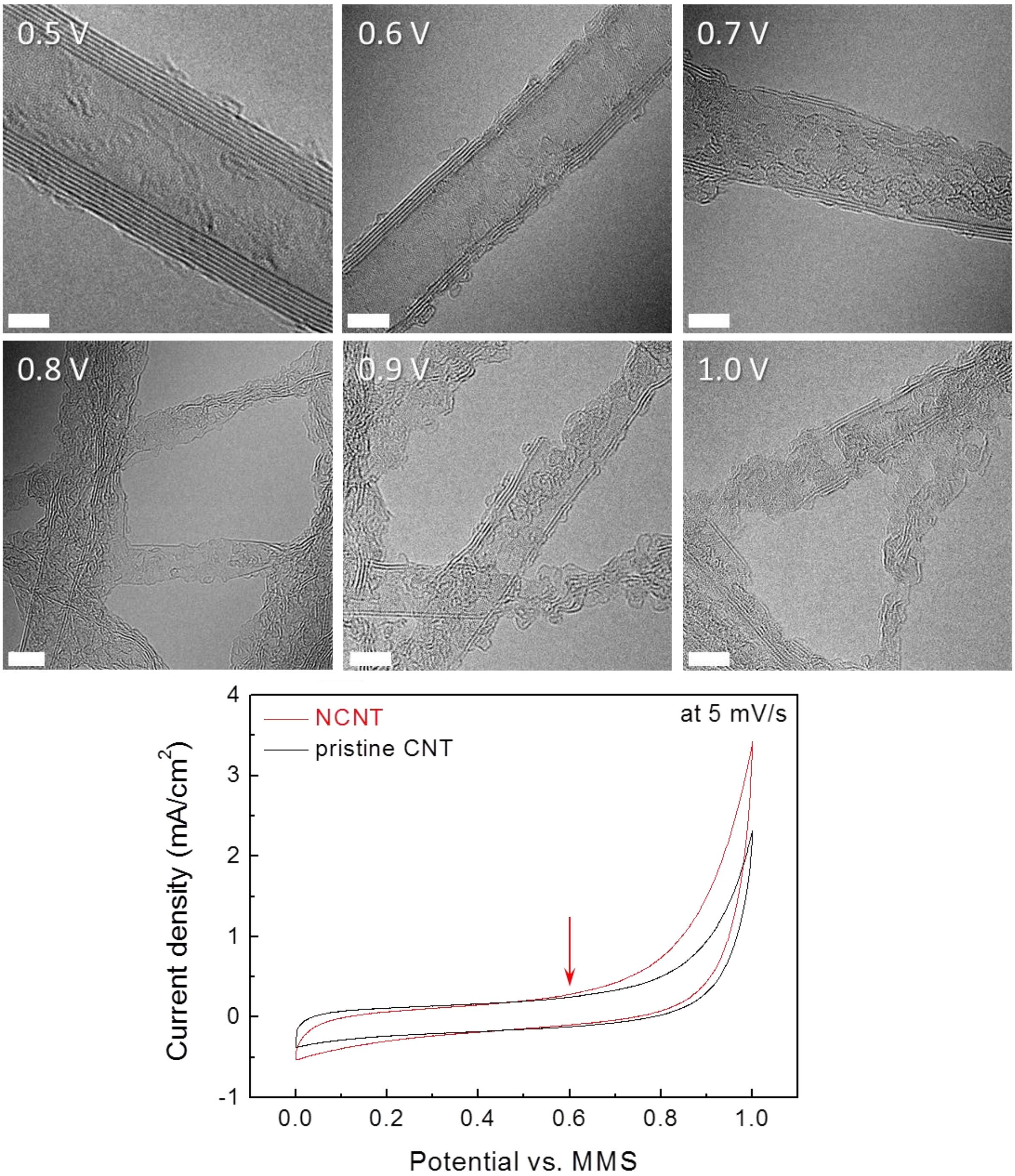

Supplementary Figure 14. N-dopant-specific unzipping at various electrical potentials
To investigate the influence of electrical potential on N-dopant-specific unzipping, unzipping process was carried out with NCNTs at various electrical potentials from 0.5-1.0 V *vs*. MMS in 1 M H_2SO_4 solution for 8 hours, and TEM images for the each potential condition was collected. In the cyclic voltammogram of NCNTs, the noticeable increase of oxidation current was observed near 0.6 V (red arrow) at which NCNTs start to be unzipped (TEM images at 0.6 V). Above 0.8 V, the oxidation current is drastically increased due to the random occurrence of vigorous oxidation reaction over NCNT surface, which is closely relevant to

uncontrollable cutting of CNT walls (TEM images at 0.9 and 1.0 V). In the case of pristine CNTs, the oxidation current starts to increase at a potential significantly higher than 0.6 V.

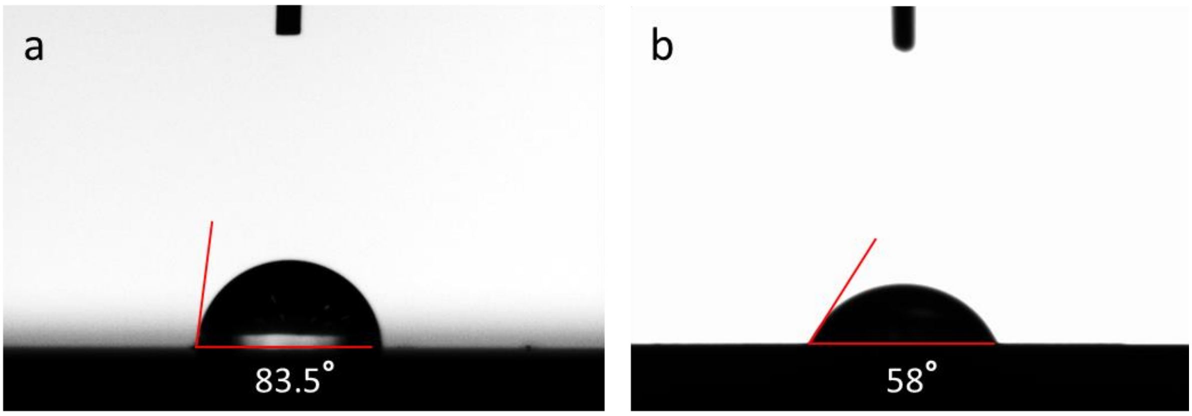

Supplementary Figure 15. **Contact angle measurements before and after unzipping**
a and **b** show contact angle of NCNT forest (83.5°) and unzipped nanostructure forests (58°), respectively.

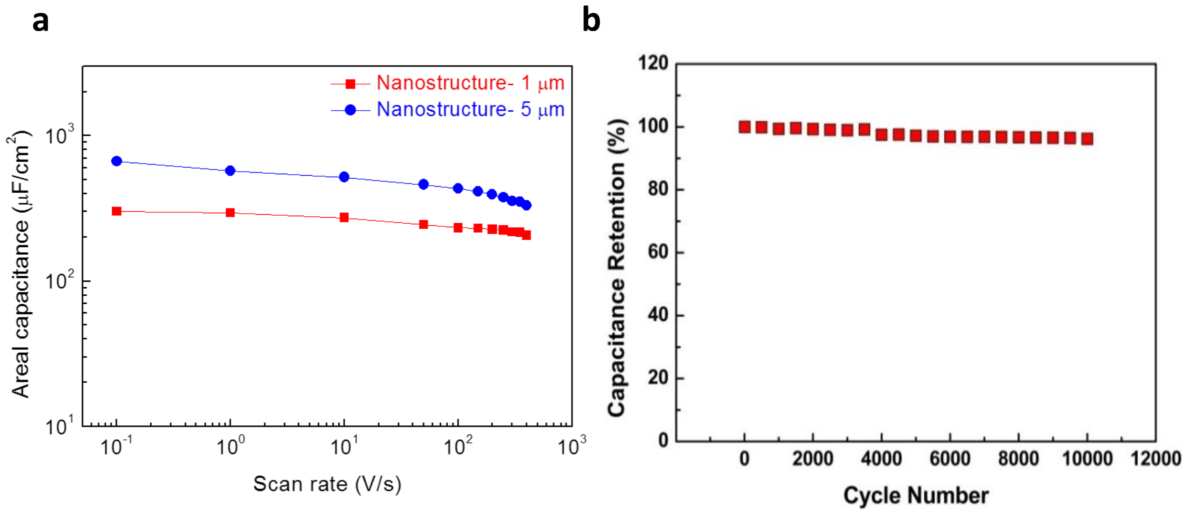

Supplementary Figure 16. Electrochemical performance of unzipped nanostructure-based DLCs.

a shows evolution of the specific capacitance of the unzipped nanostructure-based DLCs as a function of the scan rate. **b** shows excellent cycle stability of the 1-μm-unzipped nanostructure-based DLC. The cell capacitance (in F cm^{-2}) was calculated from the voltammetric discharge integrated from the cyclic voltammetry measurements over the entire potential range using the following equation:

$$C = \frac{Q}{\Delta E \times S} \quad (1)$$

where Q is the charge (in C or A.s); ΔE is the potential window (in V); S is the area (in cm^{-2}).

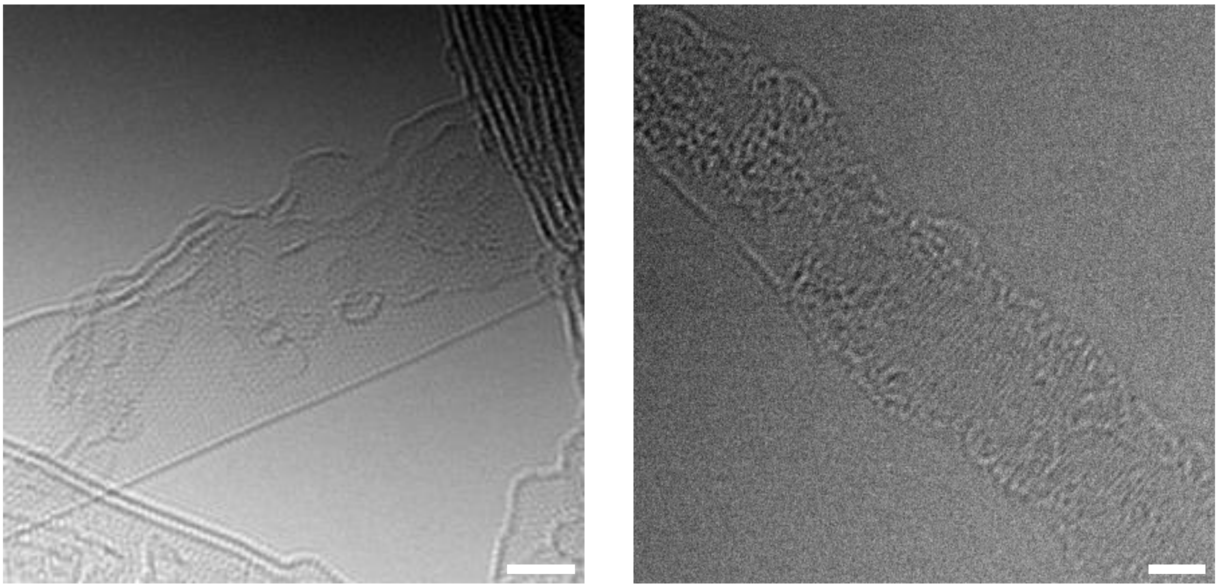

Supplementary Figure 17. TEM images of unzipped structure produced from single walled carbon nanotubes. Scale bar is 2 nm.

N_{total}	N_Q (quaternary N)	N_p (pyridinic N)	N-O bonding	Fe	Ratio of N_p : Fe
1.83 %	1.27 %	0.47 %	0.09 %	0.12 %	3.92:1
3.40 %	1.82 %	1.29 %	0.29 %	0.32 %	4.03:1
4.10 %	2.18 %	1.66 %	0.26 %	0.48 %	3.81:1

Supplementary Table 1. Estimation of atomic ratio of N_p to Fe in NCNTs at different doping levels.

Supplementary Note 1. The reaction energetics of Fe extraction from FeN_4 moiety in NCNT

In our proposed unzipping mechanism, the FeN_4 moiety in porphyrinic NCNTs[4] is the initiation site for unzipping process. In an acidic aqueous environment, two protons ($2H^+$) can replace the Fe^{2+} ion with forming the H_2N_4 moiety, represented by $FeN_4(s) + 2H^+(aq) \leftrightarrow N_4H_2(s) + Fe^{2+}(aq)$ (see Supplementary Fig. 6). The reaction energetics depend on the proton content or pH, represented by

$$\Delta G = E(N_4H_2) + \mu^{exp}(Fe^{2+}) - E(FeN_4) - 2\mu^{exp}(H^+) = -1.86 \text{ eV} - 2\mu^{exp}(H^+), \quad (2)$$

where E is DFT total energy, and μ^{exp} is experimental chemical potential[5]. As $\mu^{exp}(H+)$ varies from 0 (pH = 1) to -0.83 eV (pH = 14)[6], the reaction energy will be more negative at pH = 1 than at pH = 14. Therefore, the Fe^{2+} extraction is highly probable in an acidic condition (Figure 2c), which is consistent with the experimental Fe2p XPS measurement results.

Supplementary References

1 Kosynkin, D. V. *et al.* Longitudinal unzipping of carbon nanotubes to form graphene nanoribbons. *Nature* **458**, 872-876 (2009).

2 Dos Santos, R., Perim, E., Autreto, P., Brunetto, G. & Galvao, D. On the unzipping of multiwalled carbon nanotubes. *Nanotechnology* **23**, 465702 (2012).

3 Lee, D., Lee, W., Lee, W., Kim, S. & Kim, Y.-H. Theory, Synthesis, and Oxygen Reduction Catalysis of Fe-Porphyrin-Like Carbon Nanotube. *Phys. Rev. Lett.* **106**, 175502 (2011).

4 Kim, Y.-H., Kang, J. & Wei, S.-H. Origin of Enhanced Dihydrogen-Metal Interaction in Carboxylate Bridged Cu_2-Paddle-Wheel Frameworks. *Phys. Rev. Lett.* **105**, 236105 (2010).

5 Kim, Y.-H., Kim, K. & Zhang, S. B. First-principles calculation of thermodynamic stability of acids and bases under pH environment: A microscopic pH theory. *J. Chem. Phys.* **136**, 134112 (2012).

6 Krabbenborg, S. O., Nicosia, C., Chen, P. & Huskens, J. Reactivity mapping with electrochemical gradients for monitoring reactivity at surfaces in space and time. *Nat. Commun.* **4**, 1667 (2013).
